# Adaptive immunity in cancer immunology and therapeutics

**DOI:** 10.3332/ecancer.2014.441

**Published:** 2014-07-02

**Authors:** Emma L Spurrell, Michelle Lockley

**Affiliations:** 1 Whittington Health NHS Trust, Magdala Avenue, London N19 5NF, UK; 2 Barts Cancer Institute, Queen Mary University of London Charterhouse Square, London EC1M 6BQ, UK

**Keywords:** adaptive immunity, tumour-associated antigens, CTLA4, PD-1, PDL-1, monoclonal antibody, immune tolerance

## Abstract

The vast genetic alterations characteristic of tumours produce a number of tumour antigens that enable the immune system to differentiate tumour cells from normal cells. Counter to this, tumour cells have developed mechanisms by which to evade host immunity in their constant quest for growth and survival. Tumour-associated antigens (TAAs) are one of the fundamental triggers of the immune response. They are important because they activate, via major histocompatibility complex (MHC), the T cell response, an important line of defense against tumourigenesis. However, the persistence of tumours despite host immunity implies that tumour cells develop immune avoidance. An example of this is the up-regulation of inhibitory immune checkpoint proteins, by tumours, which induces a form of self-tolerance. The majority of monoclonal antibodies in clinical practice have been developed to target tumour-specific antigens. More recently there has been research in the down-regulation of immune checkpoint proteins as a way of increasing anti-tumour immunity.

## Introduction

Tumours do not grow in isolation, but exist within a complex network of structures, cells, and chemical signals ranging from epithelial cells, stroma, blood, and lymphatic vasculature, immune cells, cytokines, and chemokines. The vast genetic alterations characteristic of tumours produce a number of tumour antigens that enable the immune system to differentiate tumour cells from normal cells. Counter to this, tumour cells have developed mechanisms by which to evade host immunity in their constant quest for growth and survival.

A typical tumour structure includes the tumour core, the invasive margin, and the surrounding stromal and lymphoid components. Within all of these is a heterogeneous immune infiltrate that can be diverse from patient to patient, as well as within different metastatic sites of a single patient. A typical tumour will contain all immune cell-types, including macrophages, dendritic cells, natural killer (NK) cells, mast cells, B cells, and T cells [including T helper 1 (T_H_1), T helper 2 (T_H_2), regulatory T cells (T_Reg_) and cytotoxic T cells]. Within this immune milieu there are components that are beneficial, as well as components that are deleterious to the patient. Histopathological analysis of tumours has identified that different immune cells may be found in different locations within a tumour. The variation in density and distribution of immune cells within tumours is thought to affect clinical outcome [1]. Although the innate immune response plays a role in anti-tumour immunity, detailed discussion is beyond the scope of this review, which will focus on the adaptive immune response.

### Adaptive immunity and the control of tumour growth

Tumour-associated antigens (TAAs) are one of the fundamental triggers of the immune response. They are important because they activate, via, major histocompatibility complex (MHC), the T cell response, an important line of defense against tumourigenesis.

TAAs that are recognised by T cells are classified in Table 1. The review is restricted to TAAs recognised by T cells as these represent the main therapeutic targets in oncology. Although TAAs arise by different mechanisms, they are all presented to T cells via MHC class I or II on antigen presenting cells. This triggers T cell activation with expression of co-stimulatory molecules and secretion of chemokines and cytokines. The effect is to drive clonal expansion of the T cell as well as to recruit other immune effector cells (including components of the innate immune system). CD4 T cells, also known as T helper cells, secrete cytokines and chemokines that regulate different aspects of the immune response. T_H_1 CD4 T cells activate CD8 T cells, favouring cellular immunity. T_H_2 CD4 T cells act on B cells, favouring humoral immunity. CD8 T cells, which are directly cytotoxic, are activated both by direct presentation of antigen, via MHC class I, or via CD4 T cell-mediated activation. Ultimately, the tumour cell is destroyed by direct cell-mediated cytotoxicity as well an indirect antibody complement-mediated cytotoxicity [2].

### Immune editing and evasion

The persistence of tumours despite host immunity implies that tumour cells develop immune avoidance. There are a number of mechanisms by which this may occur. Some tumours have been demonstrated to lose expression of MHC molecules making them unable to present tumour antigens, thus evading T cell recognition. Some tumours secrete immunosuppressive cytokines, e.g., IL-10. There are tumours that grow within their own immune-privileged site by generating physical barriers, e.g., collagen and fibrin, thus making them invisible to the immune system.

Tumours can also evade the immune response by up-regulating inhibitory molecules and inducing a form of self-tolerance [4]. Immune checkpoints are vital for maintenance of self-tolerance and protection of normal tissue from damage at the site of an immune response. Specific regulatory cells and inhibitory receptors achieve immune tolerance. Regulatory T cells (T_Reg_) are immunosuppressive. They secrete inhibitory cytokines, such as IL-10 and TGFβ, resulting in down-regulation of effector B and T cells [5]. T cells rely on co-stimulatory signals to generate an immune response. Inhibition of co-stimulatory signals helps to maintain immune tolerance. Cytotoxic T-lymphocyte associated antigen 4 (CTLA4) and programmed cell death protein 1(PD1) are both inhibitory receptors involved in down-regulation of immune responses.

There is currently a lot of interest in the down-regulation of immune checkpoint proteins as a way of increasing anti-tumour immunity. Antibodies against CTLA-4 and PD-1 have been tested in therapeutic trials, which are discussed below.

## Tumour immunology and clinical outcome

In many cancers there is a demonstrable correlation between level of immune cell infiltration and prognosis. Different populations of cells within the immune infiltrate affect prognosis in different ways. There are a lot of published data correlating prognosis with type of immune cell infiltrate. Table 2 summarises some of the data.

Most recently it has been demonstrated that the higher the proportion of tumour infiltrating lymphocytes on core biopsy of breast cancer, the greater the likelihood of a pathological complete remission following neoadjuvant chemotherapy and Herceptin, in Her2-positive breast cancer. This data was reported at the 2013 San Antonio Breast Cancer Symposium where the team demonstrated a correlation between level of tumour-infiltrating lymphocytes and immune activation as well as clinical outcome, implying a pre-existing anti-tumour immunity. They also reported an association between high expression of inhibitory receptors PD-1 and CTLA-4, and benefit from Herceptin; hypothesizing that Herceptin overcomes tumour-mediated immunosuppression [24]. They used a mouse model of Her2-positive breast cancer to demonstrate improved efficacy of Herceptin when combined with a T-cell checkpoint inhibitor, compared to Herceptin alone [24].

The positive prognostic association of high CD4 and CD8 T cell infiltration within a tumour implies a clinically relevant anti-tumour immune response. This response is subject to down-regulation by inhibitory immune cells, including T_Reg_. Therefore, tumours with high T_Reg_ infiltration may experience less of an anti-tumour immune response, hence the association with poorer clinical outcomes.

## Targeting tumour antigens with antibodies

Targeting tumour antigens with antibodies is an established therapeutic for both solid tumours and haematological malignancies. There are different categories of tumour antigens that have been targeted by monoclonal antibodies. In haematological malignancies, antibodies have been raised against cluster of differentiation (CD) markers on T and B cells. In solid tumours, targets include growth factors, e.g., epidermal growth factor receptor (EGFR) and receptor activator of nuclear factor-κB ligand (RANKL), as well as angiogenesis, e.g., vascular endothelial growth factor (VEGF).

There are several mechanisms by which antibodies cause tumour cell death. There is the direct action of the antibody, where binding of antibody to the cell causes receptor blockade. This inhibits the downstream signaling pathways within the cell, ultimately leading to apoptosis. Immune-mediated mechanisms include antibody-dependent cellular cytotoxicity (ADCC) and complement-dependent cytotoxicity (CDC), where the Fc component (rather than the antigen-binding domain) of the antibody is most important. There is also the immune modulation of T cell function via inhibition of immune checkpoint proteins by targeted antibodies, e.g., anti-CTLA4 and anti-PD1.

Using Herceptin as an example, once the antibody has bound to Her2, expressed on the surface of a breast cancer cell, it prevents the dimerization of the receptor. Receptor dimerization is necessary for the activation of downstream signaling pathways. Herceptin inhibits the MAP kinase and PI3 kinase signaling pathways as well as causing cell cycle arrest, ultimately leading to apoptosis. The Fc component of Herceptin can trigger the innate immune response, in particular, NK cells which are directly cytotoxic to the tumour cell. Internalisation of Herceptin into the cell results in degradation of the antibody and presentation of antibody proteins to T cells via MHC. T cells are not only directly cytotoxic but will recruit other immune cells, including B cells, leading to ADCC. Finally, the Herceptin–Her2 complex results in activation of the complement pathway with binding of C1q to the antibody–antigen complex, resulting in cell lysis [25].

There are antibodies that have been conjugated to a radioisotope or cytotoxic agent to deliver these directly to the tumour cell in therapeutic doses. Conjugation of a radioisotope to antibody is used as a therapeutic in lymphoma. Most recently, T-DM1 (trastuzumab-emtansine) has shown clinical benefit in Her2-positive metastatic breast cancer where trastuzumab is conjugated to an anti-microtubule agent, emtansine. Upon ligand binding, the antibody conjugate becomes internalised into the cell enabling targeted delivery of a cytotoxin. This achieves delivery of a cytotoxic dose of a therapeutic agent directly to its target, whilst sparing healthy tissue, hence minimising toxicity [26].

Table 3 summarises examples of antibodies used in clinical practice, either NICE (National Institute for Health and Care Excellence) approved or available via the Cancer Drugs Fund (government funding of drugs not yet approved by NICE, available in England).

## Targeting immune checkpoint proteins with antibodies

### Anti- CTLA4

Ipilimumab is a monoclonal antibody that blocks CTLA4 with the aim of promoting anti-tumour immunity. CTLA4 is expressed on T cells, where it serves to regulate the magnitude of the T cell response. Once the T cell receptor has bound target antigen, the co-stimulatory receptor CD28 amplifies T cell signaling. Activated T cells are not only directly cytotoxic but also act to recruit other components of both the innate and adaptive immune response.CTLA4 counteracts the activity of the CD28 receptor thus down-regulating individual T cells and preventing recruitment of other T and immune effector cells. T_Reg_ highly express CTLA4, which enhances their proliferation and immunosuppressive activity [57].

Ipilimumab has shown the greatest clinical activity in metastatic melanoma. The pivotal phase III trial demonstrating a survival benefit with ipilimumab was published in 2010. There were three arms in the trial, ipilimumab alone, ipilimumab with gp100 (melanoma cancer vaccine), and gp100 alone. The two arms treated with ipilimumab showed the same survival suggesting that the active agent was the ipilumumab. The patients treated with ipilimumab had a significant survival advantage to those treated with gp100 alone (10.1 months vs. 6.4 months, p = 0.003) [56]. Subsequent analyses have shown the survival benefits to be durable. A pooled analysis of 1,861 patients with melanoma treated with ipilimumab in 12 prospective and retrospective studies showed that 22% were still alive at 3 years, 17% were alive at 7 years, and the longest recorded survival in the database was 9.9 years [58].

The majority of toxicities experienced with ipilimumab were immune-related, mostly affecting skin and GI tract. Diarrhoea was the most common immune-related toxicity, occurring in 31% of patients. This manifested as frank colitis in some patients, requiring corticosteroids or infliximab (anti-TNF). Other immune-related events included skin rash, vitiligo, and endocrine insufficiencies (thyroid, pituitary, and adrenal) [56].

### Anti-PD-1

PD-1 is expressed on T cells, particularly T_Reg_, its expression being induced when T cells become activated. PD-1 primarily functions in peripheral tissues where T cells are exposed to the immunosuppressive PD-1 ligands, PD-L1 and PD-L2, that are expressed by tumour cells and surrounding stroma. Once PD-1 has bound one of its ligands, it functions to inhibit kinases responsible for T cell activation, except in T_Reg_ where binding of PD-1 to ligand enhances their proliferation [57, 59].

Blockade of PD-1 by anti-PD-1 antibody has been tested in phase I. The largest cohort tested included 296 patients with advanced solid tumours, including melanoma, NSCLC, renal cell carcinoma, prostate cancer, and colorectal cancer. Responses were observed in 20–25% of patients with NSCLC, renal call carcinoma, and melanoma. No responses were observed in patients whose tumour specimens were negative for PD-L1. Similar to ipilimumab, responses were durable in some patients, with response duration of a year or more [59]. This is impressive given the fact that all patients were heavily pre-treated and no longer responding to conventional therapy.

The most common treatment-related toxicities seen with anti-PD-1 were fatigue, reduced appetite, diarrhoea, nausea, rash, and pruritus. Grade 3 or 4 treatment-related adverse events occurred in 14% of patients. Like ipilimumab, anti-PD-1 was associated with immune-related toxicity including pneumonitis, vitiligo, colitis, hepatitis, hypophysitis, and thyroiditis. There were three deaths from pulmonary toxicity [59].

There is ongoing interest in targeting PD-1 in different ways, for example, targeting one of the ligands with an anti-PD-L1 antibody, as well as combining blockade of PD-1 and CTLA4 [60].

## Conclusion

The tumour microenvironment consists of a complex milieu of cells, including immune cells. Some components of the immune infiltrate exert a beneficial anti-tumour effect, while others can down-regulate host immunity, and promote tumour-immune evasion. The proportion of the different infiltrates within a tumour has been shown to affect clinical outcome.

Antibody therapy has successfully targeted tumour antigens for many years. Targeted antibodies exert their therapeutic effect not only by inhibiting the target but also by activation of the host immune system.There is now increasing interest in using antibodies to up-regulate host anti-tumour immunity with some durable results seen in early trials so far.

## Figures and Tables

**Table 1.** Classification of tumour-associated antigens that are recognised by T cells [3].

Classification of tumour antigen	Mechanism of immune activation	Example
Cancer-testis antigens	Normal expression found in spermatocytes in testis (occasionally placenta), which is an immune-privileged site. Therefore, expression elsewhere in the body triggers T cell activation.	MAGE (melanoma antigen) BAGE (B antigen) GAGE (G antigen)
Differentiation antigens	Antigen is expressed by the tumour and the normal tissue from which it arose.	CEA – expressed in embryonic tissue and over-expressed in colorectal cancer. Gp100 – expressed in melanocytes and melanoma. PSA – expressed in normal prostate and over-expressed in prostate cancer.
Over-expressed tumourassociated antigens	Level of expression in normal tissue is below the threshold for T cell activation. Over- expression by malignant cells overrides tolerance and triggers T cell activation.	Her2 – over-expressed in breast cancer. AFP – over-expressed in hepatocellular cancer and certain germ cell tumours.
Tumour-specific antigens	These arise from genetic mutations or splicing aberrations, generating a protein that is foreign to the host immune system.	Mutant K-RAS in colorectal cancer.
Fusion proteins	Chromosomal translocation in certain tumours results in fusion of distant genes and expression of an abnormal fusion protein that is foreign to the host immune system.	BCR-ABL in CML and some ALL. EML4-ALK in non-small cell lung cancer.

CEA: carcinoembryonic antigen,

PSA: prostate specific antigen,

Her2: human epidermal receptor 2,

AFP: alpha-fetoprotein,

BCR-ABL: break point cluster region-Abelson,

EML4-ALK: echinoderm microtubule-associated protein-like 4-anaplastic lymphoma kinase,

CML: chronic myeloid leukaemia,

ALL: acute lymphoblastic leukaemia.

**Table 2.** Correlation of immune cell infiltrate and clinical outcome.

Tumour Type	Immune Cell Infiltrate	Clinical Outcome
**Associated with improved prognosis**		
Melanoma	CD4 T cell infiltrate	Better survival and higher association with spontaneous tumour regression [6–8].
Breast cancer	Intra-tumoural T cell infiltrate, including CD8 T cells and Th1 CD4 T cells.	Improved survival and earlier stage disease [9–12].
Ovarian cancer	T cell infiltrate, including CD8 T cells.	Improved survival and reduction in VEGF [13–15].
Non-small cell lung cancer	CD4 and CD8 T cell infiltrate	Improved prognosis in early stage- and advanced stage disease [16, 17].
**Associated with poor prognosis**		
Breast cancer	High T_reg_	Associated with poor prognosis disease (high-tumour grade, oestrogen receptor negative, lymph node positive) and reduced disease-free and overall survival [18, 19].
Melanoma	High T_reg_	Increased recurrence rate [20].
Ovarian cancer	High T_reg_	Associated with poor prognosis [14, 21].
Non-small cell lung cancer	High T_reg_	Associated with increased risk of recurrence in resected early stage disease [22, 23].

**Table 3.** Monoclonal antibodies used in clinical practice.

	Antigen	Antibody	Clinical Use
Angiogenesis	VEGF	Bevacizumab	Poor risk ovarian cancer [27, 28], triple negative breast cancer [29, 30], renal cell carcinoma [31], colorectal cancer [32–34], NSCLC(35).
Growth Factors	EGFR	Cetuximab	Head and neck squamous cell carcinoma [36], KRAS wild-type metastatic colorectal cancer [37–40].
	ERBB2	Herceptin	Adjuvant and metastatic Her2 positive breast cancer [41–43].
	ERBB2	Pertuzumab	Metastatic Her2 positive breast cancer [44].
	RANKL	Denosumab	Bone metastases secondary to solid tumours [45].
Haemopoieitic antigens	CD20	Rituximab	Non-Hodgkin’s lymphoma [46, 47].
	CD20	Ofatumumab	Refractory CLL [48].
	CD52	Alemtuzumab	CLL [49, 50].
	Proteosome inhibitor	Bortezomib	Myeloma [51].
	CD30	Brentuximab	Relapsed Hodgkin’s lymphoma [52].
Conjugated Antibodies	CD20	^90^Y-labelled ibritumomab	Non-Hodgkin’s lymphoma [53].
	CD20	^131^I-labelled tositumomab	Non-Hodgkin’s lymphoma [54, 55].
	ERBB2	T-DM1 - ERBB2-emtansine (antibody-drug conjugate)	Metastatic Her2 positive breast cancer [26].
Immunomodulatory	CTLA4	Ipilimumab	Metastatic melanoma [56].

NSCLC: non-small cell lung cancer,

CLL: chronic lymphocytic leukaemia.
